# Cognitive–Affective Dimension After Virological Cure of Hepatitis C Virus Infection: WBSI Dynamics, Liver Status, and the Role of Comorbidities

**DOI:** 10.3390/diseases14100386

**Published:** 2026-10-21

**Authors:** Alexia Anastasia Ștefania Baltă, Alina-Maria Lescai, Valerian Ionut Stoian, Mariana Daniela Ignat, Adriana Liliana Vlad, Corina Popazu, Daniela-Ioanina Prisăcaru, Florentin Dimofte, Elena Dumea, Mihaela Dediu-Anghel, Liliana Baroiu

**Affiliations:** 1 Faculty of Medicine and Pharmacy, Medical-Pharmaceutical Research Center, “Dunarea de Jos” University of Galati, 800008 Galati, Romania; 2 Tübinger Akademie für Verhaltenstherapie, David-von-Stein-Weg 26, 72072 Tübingen, Germany; ioaninaprisacaru@gmail.com; 3 Faculty of Medicine and Pharmacy, Ovidius’ University of Constanta, 900527 Constanta, Romania; elenadumea@yahoo.com

**Keywords:** hepatitis C, direct-acting antivirals, sustained virologic response, White Bear Suppression Inventory, comorbidities, quality of life, liver fibrosis

## Abstract

Introduction: Virological cure in chronic infection with hepatitis C virus (HCV) is followed by important clinical and biological benefits, including psychological ones. The White Bear Suppression Inventory (WBSI) assesses the tendency to suppress intrusive thoughts and was used in this study as a tool to provide a cognitive–affective assessment of patients with chronic HCV infection—both at the start of antiviral therapy and five years after achieving virological cure. Objective: This study aimed to characterize patients with HCV infection who received antiviral therapy and completed the WBSI, to assess longitudinal changes in WBSI scores, and to identify clinical, biological, and comorbidity-related factors associated with these changes. Methods: The study employed an ambispective observational longitudinal cohort study design conducted on a sample of 386 patients, of whom 81 completed the WBSI both at baseline and at follow-up. Results: The WBSI subsample did not differ significantly from the remainder of the cohort in terms of age, cytolysis markers, bilirubin, albumin, platelet count, or major comorbidities. The WBSI score decreased significantly from baseline to follow-up, from 61.36 to 54.16, with a mean difference of 7.20 points (t = 6.32; *p* < 0.001). The correlation between the baseline and follow-up scores was very strong (r = 0.899; *p* < 0.001), indicating substantial interindividual stability. ΔWBSI was weakly but significantly correlated with the change in total bilirubin (r = 0.254; *p* = 0.022), with no significant correlations with the other biological markers. In the multivariable model, cardiovascular comorbidities (B = −8.469; *p* < 0.001) and metabolic comorbidities (B = −9.869; *p* = 0.002) were independent negative predictors of WBSI scoring, whereas fibrosis evolution, age, sex, and baseline total bilirubin had no significant influence. Conclusions: Improvements in WBSI scores observed only in specific patient subgroups, one year after achieving HCV virological cure, underscore the need to integrate psychological assessment into long-term monitoring, particularly for patients with cardiovascular and metabolic comorbidities.

## 1. Introduction

Over the past decade, the treatment of chronic HCV infection has been revolutionized by the use of direct-acting antivirals, which have transformed a progressive chronic disease into a condition that is frequently curable from a virological standpoint. Achieving susteined viral response(SVR) is associated with improvements in hepatic biomarkers, reduced inflammation, and, in some patients, regression of noninvasive fibrosis markers [1,2,3]. However, virological cure does not always equate to complete normalization of clinical or psychological status.

Previous studies assessing patient-reported outcomes after HCV treatment have predominantly focused on broad health-related quality of life, functional status, fatigue, anxiety, and depression. Improvements following viral clearance have been documented using generic or disease-specific quality-of-life instruments, although residual impairment may persist in patients with advanced liver disease, social vulnerability, substance-use-related factors, or other comorbidities [4,5,6,7,8]. In contrast, the White Bear Suppression Inventory (WBSI) does not measure global health-related quality of life or diagnose anxiety or depression. It specifically assesses the tendency to suppress unwanted thoughts, thereby capturing a cognitive-regulatory dimension that is conceptually distinct from the broader outcomes evaluated in previous HCV studies [9,10]. The present analysis therefore aims to complement, rather than reproduce, the existing post-SVR quality-of-life literature.

The traditional paradigm, centered solely on viral eradication, transaminase levels, and fibrosis, does not fully capture the patient’s post-treatment experience. HCV infection is associated with extrahepatic and neuropsychiatric manifestations that affect fatigue, mood, quality of life, and cognitive functioning [11,12,13,14]. In addition, post-SVR studies show that psychological benefits are real for some patients, but are not uniform [15,16,17,18,19,20,21].

Within this framework, the WBSI provides a useful measure of the tendency to suppress intrusive thoughts. In the case of chronic diseases, assessing a thought suppression score helps clarify how the patient processes the diagnosis, the residual risk, and the uncertainty associated with long-term monitoring [22,23].

The WBSI is a 15-item self-report instrument designed to assess the tendency to suppress unwanted thoughts. Each item is rated on a 5-point Likert scale ranging from 1 (strongly disagree) to 5 (strongly agree), yielding a total score between 15 and 75, with higher scores indicating a greater tendency toward thought suppression and intrusive thinking. The WBSI is not a diagnostic instrument for anxiety, depression, or overall mental health. For the longitudinal analysis, ΔWBSI was calculated as baseline minus follow-up score; therefore, positive values indicate a reduction in WBSI score, whereas negative values indicate an increase.

The present study analyzes WBSI dynamics in patients with HCV who received antiviral treatment and seeks to integrate these parameters with liver status, biological markers, and comorbidity burden. The working hypothesis was that, post-SVR, WBSI parameters do not depend exclusively on fibrosis evolution or biochemical normalization, but are modulated by systemic comorbidities and individual vulnerability.

## 2. Materials and Methods

### 2.1. Study Design and Analyzed Population

The study employed a retrospective/prospective observational longitudinal cohort conducted on a sample of 714 patients, of whom 81 completed the WBSI both at baseline and at follow-up, who underwent antiviral treatment for HCV between 2017 and 2025 in the Second Infectious Diseases Clinic of the “Sf. Cuvioasa Parascheva” Clinical Infectious Diseases Hospital in Galați. The biochemical follow-up evaluation, the follow-up Fibroscan and the application of the White Bear Suppression Inventory (WBSI) questionnaire were performed on the same day, at the initial assessment, at the start of antiviral therapy and at the control carried out 60 months after the start of therapy. The study was approved by the Ethics Committee of the “Dunărea de Jos” University of Galați, no. 42426 from 12 April 2024, and all patients signed informed consent before inclusion in the analysis. Patients predominantly had HCV genotype 1 infection, accounting for 99% of circulating strains in Romania [14], and were of white race, Romanian ethnicity and received direct acting antiviral therapies (DAA) with various regimens according to the indications of the national program at the time of therapy initiation, namely: ombitasvir/paritaprevir/ritonavir and dasabuvir, ledipasvir/sofosbuvir, elbasvir/grazoprevir, sofosbuvir/velpatasvir, or glecaprevir/pibrentasvir [24].

**Figure 1:**
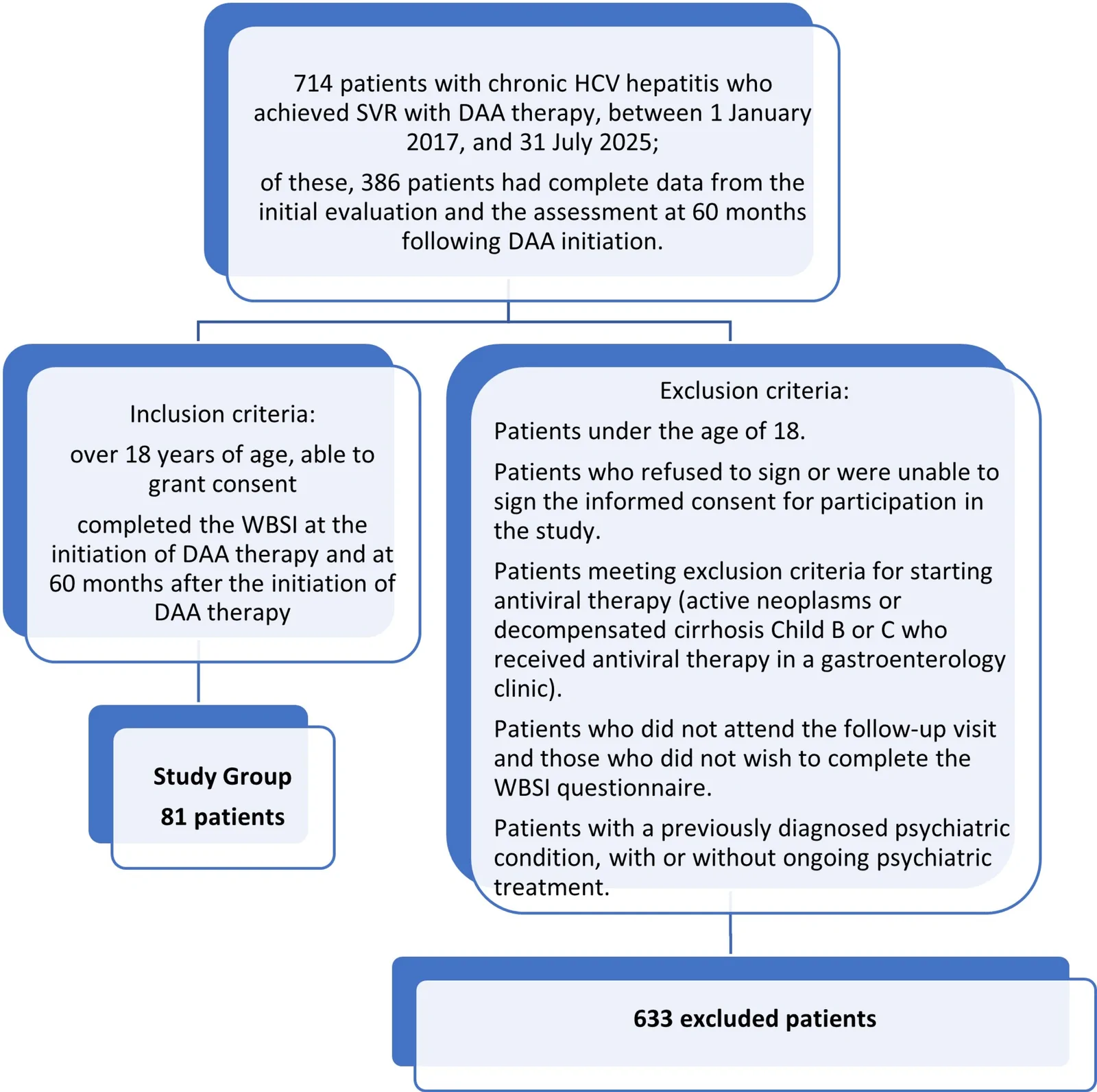
Patient group selection.

A longitudinal analysis was conducted on a cohort of patients with chronic HCV infection and complete follow-up after antiviral therapy. The psychological component was assessed in a subsample of 81 patients who completed the WBSI at baseline and follow-up. The reference cohort used to assess the representativeness of the subsample included 386 patients with available clinical and biological data (Figure 1).

To reduce the risk of interpretation influenced by selection bias, patients who completed the WBSI were compared with those who did not complete the instrument. Age at treatment initiation, cytolysis markers, total bilirubin, albumin, platelet count, and the main categories of comorbidities were analyzed.

### 2.2. Psychological Instrument

The White Bear Suppression Inventory was used to quantify the tendency to suppress intrusive thoughts. Higher scores indicate a less favorable cognitive–affective burden. For the longitudinal analysis, ΔWBSI was calculated as the difference between the baseline and follow-up scores; thus, positive values indicate score improvement, whereas negative values indicate worsening.

### 2.3. Clinical and Biological Variables

The analysis included demographic variables, markers of hepatic cytolysis (aspartate aminotransferase-AST, alanine aminotransferase-ALT), indicators of liver function and synthetic capacity (bilirubin, international normalized ratio- INR, albumin), indirect hematological parameters of portal hypertension/fibrosis (platelet count), the evolution of liver fibrosis, and cardiovascular, metabolic, and endocrine comorbidities. Changes in biological markers were analyzed as differences between baseline and follow-up values.

### 2.4. Statistical Analysis

Group comparability was assessed using independent-samples *t*-tests for continuous variables and Chi-square/Fisher’s exact tests for categorical variables. WBSI dynamics were analyzed using a paired-samples *t*-test. The relationship between ΔWBSI and fibrosis evolution was tested using one-way ANOVA, with homogeneity of variances assessed by Levene’s test and Bonferroni post hoc comparisons. Associations between ΔWBSI and changes in biological markers were evaluated using Pearson correlations. Independent predictors of ΔWBSI were analyzed using multivariable linear regression with the Enter method.

## 3. Results

### 3.1. Representativeness of the WBSI Subsample

The WBSI subsample included 81 patients. Although patients with a previously diagnosed psychiatric disorder were excluded, no standardized baseline psychiatric assessment was performed; therefore, subclinical or undiagnosed anxiety, depression, or other psychological conditions cannot be excluded. Comparison of this subgroup with patients who did not complete the WBSI questionnaire revealed no major imbalances in the demographic, or clinical and biological variables assessed. Mean age, baseline transaminase levels, total bilirubin, albumin, and platelet count were similar between groups, and inferential tests indicated no statistically significant differences. The distribution of cardiovascular, metabolic, and endocrine comorbidities was also comparable. Although questionnaire completion was numerically more frequent among patients with endocrine comorbidities, the difference was not statistically significant. Overall, these results indicate a low risk of selection bias for the psychological subsample with respect to the variables assessed (Table 1).

Regarding chronic HCV infection, we included Caucasian patients with all stages of fibrosis—including compensated liver cirrhosis (Child–Pugh A)—and infection durations ranging from 6 months to over 20 years; these patients had either previously received interferon therapy (without achieving virological cure) or were treatment-naive, yet all presented with positive HCV RNA. All patients included in the present longitudinal analysis had achieved SVR after DAA therapy. HCV-associated extrahepatic manifestations observed in the study group included chronic urticaria and chronic arthropathies, both of which improved following antiviral treatment.

### 3.2. Longitudinal Dynamics of the WBSI Score

The mean WBSI score decreased from 61.36 at baseline to 54.16 at follow-up, suggesting an overall improvement in the cognitive–affective dimension. The paired-samples *t*-test demonstrated a significant mean reduction of 7.20 points (*p* < 0.001). At the same time, the correlation between the baseline and follow-up scores was very strong (r = 0.899; *p* < 0.001), indicating that the relative ranking of individuals remained stable. The ΔWBSI values showed wide individual dispersion, ranging from −37 to 30, highlighting different psychological trajectories of improvement, stability, or worsening (Table 2).

### 3.3. ΔWBSI and the Evolution of Liver Fibrosis

The evolution of liver fibrosis was analyzed in relation to ΔWBSI using one-way ANOVA. Mean ΔWBSI values were higher in the stable and progressive fibrosis groups than in the regressive fibrosis group; however, the regressive group showed much greater variability. Levene’s test indicated unequal variances (*p* < 0.001), warranting caution in interpreting the ANOVA result. The overall difference among fibrosis-evolution categories was statistically significant (F = 3.66; *p* = 0.030). Bonferroni post hoc analysis identified a significant difference between the regressive and stable groups (mean difference = −6.19; *p* = 0.040), whereas the other pairwise comparisons were not significant. These findings should therefore be interpreted as an exploratory association rather than a direct causal relationship (Table 3).

### 3.4. ΔWBSI and Changes in Biological Markers

Correlations between ΔWBSI and changes in biological markers showed that cognitive–affective recovery is not uniformly explained by the dynamics of conventional hepatic parameters. The only significant association was observed with the change in total bilirubin, and the strength of this relationship was weak (r = 0.254; *p* = 0.022). No significant correlations were identified with ΔAST, ΔALT, ΔINR, ΔALB, or ΔPLT, indicating that biological improvement does not automatically translate into a proportional reduction in the suppression of intrusive thoughts. This dissociation supports a biopsychosocial interpretation of post-SVR recovery (Table 4).

### 3.5. Influence of Comorbidities on ΔWBSI

The comorbidity burden was associated with meaningful differences in WBSI score change. The comorbidity profile of the patients included in the study revealed the following: the patients had no psychiatric history and had not used antidepressants, anxiolytics, or psychological therapy prior to or during antiviral treatment; 13 patients (16.04%) had no comorbidities, 37 (45.67%) had one comorbidity, and 31 (38.27%) had more than two types of comorbidities; 41 patients (50.61%) had cardiovascular comorbidities (including arterial hypertension), 21 (25.92%) had endocrine comorbidities, and 12 (14.81%) had metabolic comorbidities (including diabetes mellitus, dyslipidemia, and obesity). Patients without comorbidities and those with a single comorbidity showed greater improvement, whereas patients with two or more comorbidities exhibited less improvement and greater variability. Variance heterogeneity was significant (Levene *p* < 0.001), and ANOVA indicated differences among the comorbidity-burden categories (F = 3.888; *p* = 0.025). The Bonferroni post hoc analysis showed a significant difference between patients with one comorbidity and those with two or more comorbidities (*p* = 0.042).

Cardiovascular comorbidities had the most evident impact in the comparative analysis. Patients without cardiovascular pathology showed a substantially greater improvement in WBSI score than those with cardiovascular comorbidities; the unequal-variance comparison remained significant (*p* = 0.002). For metabolic comorbidities, the mean difference was clinically important but did not reach statistical significance after adjustment for heterogeneity of variances (*p* = 0.064). Endocrine comorbidities were not associated with significant differences in ΔWBSI (*p* = 0.570) (Table 5).

### 3.6. The Multivariate Model and the Exploratory WBSI–Comorbidity Score

The multivariate linear regression model included the variables considered clinically and statistically relevant: fibrosis progression, sex, age at treatment initiation, baseline total bilirubin, and cardiovascular and metabolic comorbidities. The model demonstrated moderate explanatory capacity (R = 0.54; R^2^ = 0.30; adjusted R^2^ = 0.24) and was statistically significant overall (F(6,74) = 5.20; *p* < 0.001).

Cardiovascular and metabolic comorbidities were the only significant independent predictors. Both had negative coefficients, indicating a smaller improvement in WBSI score among patients with these comorbidities after adjustment for the other factors. Fibrosis progression, age, sex, and baseline total bilirubin did not remain significant in the model. The residual distribution supported the adequacy of the model, with a mean residual of 0.00 and standardized residuals ranging from −3.31 to 2.81 (Table 6).

**Figure 2:**
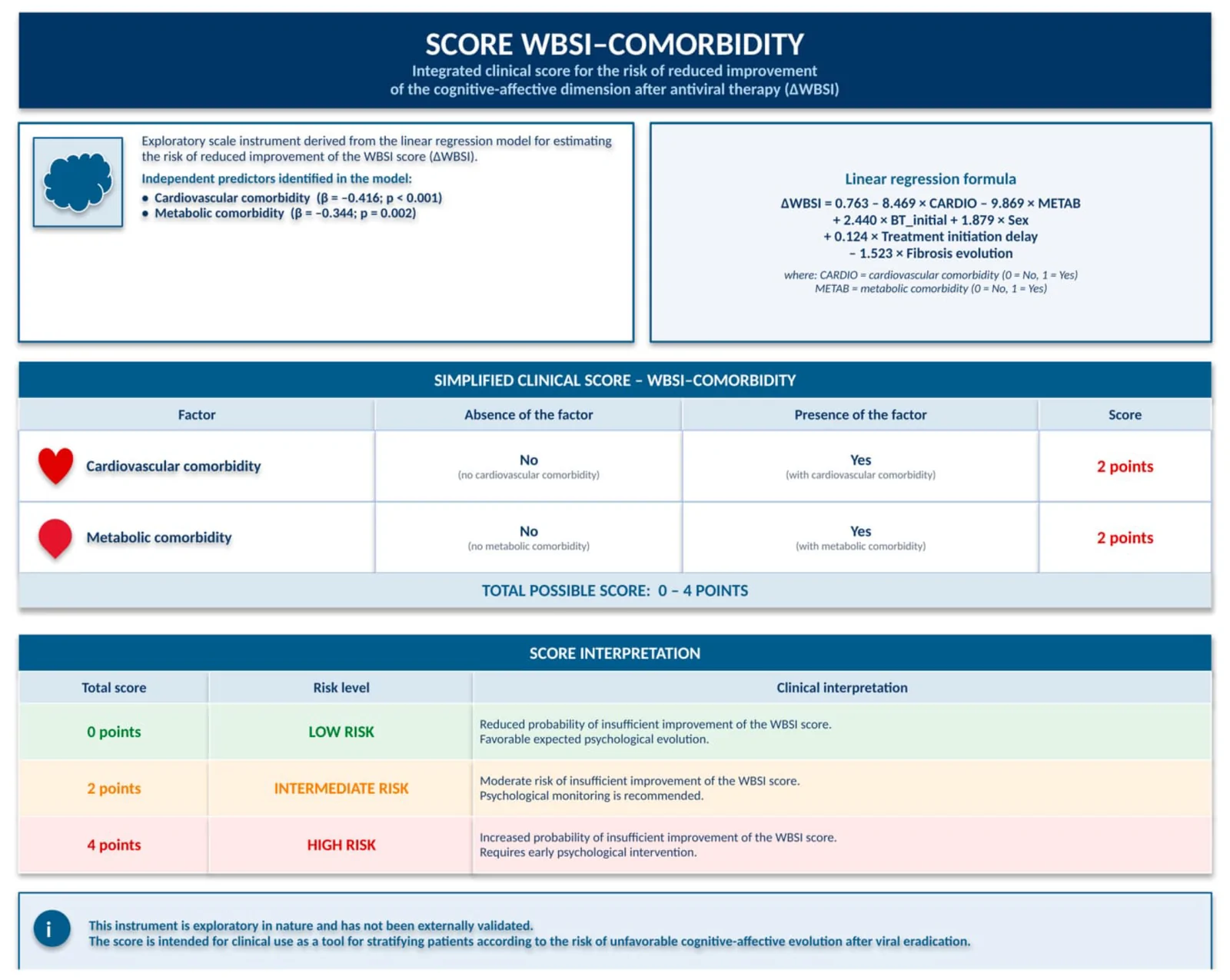
Exploratory scalar instrument derived from the multivariable linear regression model, designed to assess the risk of an unfavorable cognitive–affective trajectory in patients with HCV after antiviral therapy.

Based on the independent predictors identified, an exploratory WBSI–comorbidities clinical score was proposed, aimed at stratifying patients according to their risk of limited psychological improvement after SVR. The instrument is preliminary in nature and requires external validation before broad clinical use. The score integrates the main independent predictors identified in the analysis, namely cardiovascular and metabolic comorbidities, to which points are assigned in proportion to their negative impact on the change in the WBSI score (Figure 2).

The proposed scoring model (WBSI–comorbidities) is indicative and requires future validation; The proposed WBSI–comorbidity score should be regarded solely as a hypothesis-generating exploratory tool. It is not intended for clinical decision-making in its current form and requires external validation before its potential clinical utility can be assessed. The study results justify the need for a comprehensive psychological assessment of these patients, including evaluations of anxiety, depression, quality of life, and the perceived impact of chronic diseases on general health status.

## 4. Discussion

This study highlights that the cognitive–affective dimension of recovery after antiviral treatment for HCV can improve, but is not entirely dependent on hepatic dynamics. The significant decrease in the WBSI score indicates a reduction in the psychological burden after SVR, whereas the very strong correlation between the baseline and follow-up scores demonstrates the persistence of a stable individual component.

The WBSI has been applied in psychological and behavioral research beyond traditional psychiatric populations. For example, Soetens et al. used the instrument in restrained eaters and found higher levels of thought suppression and intrusive thinking among disinhibited restrained eaters [9]. This supports the usefulness of the WBSI for identifying maladaptive cognitive-regulatory patterns in health-related contexts. Nevertheless, the WBSI should not be interpreted as a global mental-health or quality-of-life measure. It captures a relatively specific tendency toward suppression of unwanted thoughts and, depending on its factor structure, aspects of intrusive thinking.

The results are consistent with the literature describing the benefits of SVR for quality of life and mental health, while also noting the persistence of vulnerabilities in certain subgroups [12,13,14,15,16,17,18]. Thus, viral cure may reduce the uncertainty and stress associated with chronic infection, but it does not automatically eliminate the cognitive mechanisms formed during the course of the disease or the influence of persistent comorbidities.

An important finding is the absence of a linear relationship between fibrosis regression and WBSI improvement. In the univariate analysis, ΔWBSI differed across categories of fibrosis evolution; however, the direction of the effect did not support a simple parallel between structural regression and psychological recovery. In the multivariate model, fibrosis evolution did not remain an independent predictor. This finding suggests that patients’ perception of illness and prognosis may be more strongly influenced by overall health status than by an isolated change in fibrosis score.

Cardiovascular and metabolic comorbidities had the most consistent role. They independently limited WBSI improvement, suggesting that systemic somatic burden may maintain an increased level of stress, health-related concern, and cognitive–affective vulnerability. The relationship between cardiovascular disease, anxiety, depression, and quality of life has been described in the general literature [25,26,27], and the association between metabolic syndrome, quality of life, and anxiety is supported by systematic reviews and meta-analyses [28,29].

The weak correlation between ΔWBSI and the change in total bilirubin indicates a possible specific link between hepatic function and psychological status, but the absence of correlations with the other biological markers shows that this mechanism is limited. Cognitive–affective recovery should therefore be interpreted within a biopsychosocial model, in which hepatic markers, residual risk, comorbidities, social support, illness experience, and coping strategies contribute simultaneously.

The originality of this analysis lies in the integration of the WBSI into a cohort of patients with HCV after antiviral therapy and in the proposal of an exploratory score based on comorbidities. Although this score has not yet been externally validated, it translates the regression results into a practical instrument for preliminary stratification and may guide differentiated psychological monitoring.

The limitations of the study are:

(1) First, the psychological analysis was based on a subsample of 81 patients, which limits statistical power, particularly for small subgroup comparisons. Although the WBSI subsample was comparable with the remaining complete-data cohort for the measured variables, selection bias related to unmeasured characteristics cannot be excluded. Second, the study was conducted at a single center in Romania in a geographically and ethnically homogeneous population; therefore, the generalizability of the findings to populations with different racial, ethnic, cultural, and healthcare backgrounds is limited. Third, all patients included in the longitudinal analysis achieved SVR. The absence of a non-SVR or otherwise appropriate comparison group prevents the attribution of the observed WBSI changes specifically to virological clearance, because temporal effects, repeated assessment, treatment-related factors, or unmeasured changes occurring during follow-up cannot be excluded. Fourth, although patients with known psychiatric diagnoses were excluded, no comprehensive standardized assessment of anxiety, depression, or other psychiatric symptoms was performed; therefore, undiagnosed or subclinical psychological conditions may have influenced WBSI trajectories. Finally, variance heterogeneity affected several subgroup comparisons, and the exploratory WBSI–comorbidity score was derived and tested in the same small sample. It should therefore be considered hypothesis-generating and requires independent external validation, calibration, and assessment of predictive performance before any clinical application.

Although the subsample was comparable with the rest of the cohort for the available variables, differences related to unassessed factors, such as socioeconomic status, education and social support cannot be excluded.

(2) Some analyses were affected by the heterogeneity of variances, which requires cautious interpretation.

Virological cure achieved through direct-acting antiviral therapies is consistently linked to a clear improvement in both the prognosis and the psychological well-being of patients with chronic disease. A subset of these patients is diagnosed and treated at advanced stages of hepatic fibrosis or presents with additional liver-related comorbidities; they remain under close monitoring to ensure the early detection and treatment of complications such as liver cirrhosis and hepatocellular carcinoma. Psychological assessment and support are crucial components of the long-term monitoring pathway for these patients. In our clinical practice, the use of the WBSI questionnaire has proven highly valuable, demonstrating that psychological evaluation following the achievement of a sustained virological response can significantly enhance a patient’s quality of life.

## 5. Conclusions

Patients with HCV after antiviral therapy showed a significant decrease in WBSI score. Hepatic biological markers explained only part of the evolution of WBSI, whereas cardiovascular and metabolic comorbidities were the main independent predictors of reduced improvement. Therefore, post-SVR monitoring should include not only hepatic assessment but also the identification of psychological vulnerability, particularly in patients with a systemic comorbid burden.

Integrating the WBSI and a comprehensive psychological evaluation into the assessment of patients with HCV may contribute to a multidimensional approach to post-therapeutic recovery and to the identification of subgroups that may benefit from psychological support or the integrated management of comorbidities.

## Figures and Tables

**Table 1:** Comparison of demographic, clinical–biological, and comorbidity variables according to WBSI questionnaire completion status.

Variable	WBSI Completed (n = 81)	WBSI Not Completed (n = 305)	*p*
Age at treatment initiation, years	59.89 ± 9.78	60.37 ± 11.67	0.705
AST—baseline, U/L	46.69 ± 15.09	45.79 ± 15.60	0.643
ALT—baseline, U/L	51.19 ± 20.19	51.30 ± 20.95	0.965
Total bilirubin—baseline, mg/dL	0.66 ± 0.20	0.68 ± 0.21	0.641
Albumin—baseline, g/dL	3.66 ± 0.29	3.73 ± 0.31	0.069
Platelets—baseline, ×1000/µL	209.42 ± 58.35	223.23 ± 62.65	0.075
Cardiovascular comorbidities, n (%)	41 (50.6%)	167 (54.8%)	0.507
Metabolic comorbidities, n (%)	12 (14.8%)	52 (17.0%)	0.631
Endocrine comorbidities, n (%)	21 (25.9%)	55 (18.0%)	0.112

Continuous variables are presented as mean ± standard deviation. *p* values correspond to the appropriate independent-samples *t*-test or Chi-square test. None of the between-group differences reached statistical significance.

**Table 2:** Longitudinal dynamics of the WBSI score between baseline and follow-up.

Measure	n	Mean ± SD	Range	Statistical Result
WBSI—baseline total score	81	61.36 ± 23.32	—	—
WBSI—follow-up total score	81	54.16 ± 20.12	—	—
ΔWBSI (baseline—follow-up)	81	7.20 ± 10.24	−37 to 30	95% CI: 4.93 to 9.46; t(80) = 6.32; *p* < 0.001
Baseline vs. follow-up correlation	81	—	—	r = 0.899; *p* < 0.001

Positive ΔWBSI values indicate a reduction in WBSI score at follow-up; negative values indicate worsening.

**Table 3:** ΔWBSI according to the evolution of liver fibrosis.

Fibrosis Evolution	N	ΔWBSI, Mean ± SD	95% CI for Mean
Regressive	48	4.73 ± 12.45	1.11 to 8.35
Stable	25	10.92 ± 3.99	9.27 to 12.57
Progressive	8	10.38 ± 0.74	9.75 to 11.00

Levene statistic = 9.39, *p* < 0.001; one-way ANOVA F(2,78) = 3.66, *p* = 0.030. Bonferroni: regressive vs. stable, mean difference = −6.19, *p* = 0.040; regressive vs. progressive, *p* = 0.420; stable vs. progressive, *p* = 1.000.

**Table 4:** Correlation between ΔWBSI and changes in biological markers during the follow-up period.

Biological Marker Change	Pearson Correlation with ΔWBSI (r)	*p*
ΔAST	−0.026	0.816
ΔALT	−0.133	0.236
Δ total bilirubin	0.254	0.022
Δ direct bilirubin	0.071	0.529
ΔINR	0.050	0.660
Δ albumin	0.112	0.320
Δ platelets	0.117	0.297

n = 81 for all correlations. The correlation with Δ total bilirubin was statistically significant at the 0.05 level.

**Table 5:** Variation in ΔWBSI according to comorbidity burden and major comorbidity categories.

Analysis	Group	N	ΔWBSI, Mean ± SD	Statistical Result
Number of comorbidities	None	13	10.23 ± 0.60	ANOVA F = 3.888; *p* = 0.025; Levene *p* < 0.001
	One	37	9.38 ± 5.56	One vs. ≥2: *p* = 0.042 (Bonferroni)
	Two or more	31	3.32 ± 14.74	
Cardiovascular comorbidity	No	40	10.8 ± 3.2	Welch t = 3.35; *p* = 0.002
	Yes	41	3.7 ± 13.2	Mean difference = 7.12; 95% CI 2.84 to 11.39
Metabolic comorbidity	No	69	8.7 ± 7.7	Welch t = 2.05; *p* = 0.064
	Yes	12	−1.7 ± 17.3	Mean difference = 10.41; 95% CI −0.69 to 21.50
Endocrine comorbidity	No	60	7.6 ± 8.8	t = 0.57; *p* = 0.570
	Yes	21	6.1 ± 13.8	Mean difference = 1.49; 95% CI −3.70 to 6.68

Because variance heterogeneity was present in several comparisons, unequal-variance test results were used where appropriate and subgroup findings should be interpreted cautiously.

**Table 6:** Multivariable linear regression model for change in WBSI score (ΔWBSI).

Predictor	B	Std. Error	Beta	t	*p*
Cardiovascular comorbidities	−8.469	2.152	−0.416	−3.936	<0.001
Metabolic comorbidities	−9.869	3.067	−0.344	−3.218	0.002
Total bilirubin—baseline, mg/dL	2.440	5.342	0.049	0.457	0.649
Sex	1.879	2.611	0.078	0.719	0.474
Age at treatment initiation	0.124	0.106	0.118	1.160	0.250
Fibrosis progression	−1.523	3.551	−0.045	−0.429	0.669

Model summary: R = 0.54; R^2^ = 0.30; adjusted R^2^ = 0.24; standard error of the estimate = 8.93; F(6,74) = 5.20; *p* < 0.001. Residual range: −29.58 to 25.08; standardized residual range: −3.31 to 2.81.

## Data Availability

Data Availability Statements are available on request through the corresponding authors.
